# Microglial SLC25A28 Knockout Mitigates Spinal Cord Injury in Mice by Inhibiting Heme Synthesis and Subsequent NOX2 Activation

**DOI:** 10.1111/cns.70638

**Published:** 2025-11-02

**Authors:** Huangtao Chen, Shaochun Guo, Yanglan Mi, Ruili Han, Yuxin Xi, Tingwei Peng, Longhui Fu, Weidong Liu, Ruiyu Ma, Beibei Yu, Yongfeng Zhang, Luyao Li, Jing Ye, Shouping Gong

**Affiliations:** ^1^ Department of Neurosurgery The Second Affiliated Hospital of Xi'an Jiao Tong University Xi'an Shaanxi China; ^2^ Department of Neurosurgery Tangdu Hospital, the Fourth Military Medical University Xi'an China; ^3^ State Key Laboratory of Cancer Biology, Department of Pathology Xijing Hospital and School of Basic Medicine, Fourth Military Medical University Xi'an Shaanxi China; ^4^ Xi'an No. 9 Hospital Xi'an Shaanxi China; ^5^ Department of Anesthesiology Tangdu Hospital, Fourth Military Medical University Xi'an Shaanxi China; ^6^ Department of Cardiology Tangdu Hospital, Fourth Military Medical University Xi'an Shaanxi China; ^7^ Department of Foot and Ankle Surgery Honghui Hospital of Xi'an Jiaotong University Xi'an Shaanxi China; ^8^ Xi'an Medical University Xi'an Shaanxi China

**Keywords:** heme, microglia, mitochondrial iron, neuroinflammation, NOX2, SLC25A28, spinal cord injury

## Abstract

**Background:**

Microglial overactivation‐driven neuroinflammation exacerbates secondary damage after spinal cord injury (SCI), but the role of mitochondrial iron metabolism in this process is not well understood. This study investigates the function of the mitochondrial iron transporter solute carrier family 25 member 28 (SLC25A28) in post‐SCI neuroinflammation.

**Methods:**

Microglia‐specific SLC25A28 knockout (A28‐MGKO) mice were generated by crossing SLC25A28^flox/flox^ mice with Cx3cr1‐CreERT2 mice and subjected to clip‐compression spinal cord injury (SCI) at the T9 level. Motor recovery was evaluated using the Basso Mouse Scale (BMS), while histological and biochemical assessments including hematoxylin–eosin and Nissl staining, Iba1 immunohistochemistry, Evans blue permeability, and tissue water content were performed to evaluate lesion severity, neuronal survival, microglial activation, and blood–spinal cord barrier integrity. In vitro, primary microglia isolated from A28‐MGKO mice and BV2 cells with SLC25A28 overexpression were used to investigate mitochondrial iron homeostasis, heme biosynthesis, and NOX2‐mediated oxidative stress. Mitochondrial iron content was quantified using a ferrozine‐based assay and Mito‐FerroGreen staining, while ROS production, cytokine release, and inflammatory signaling were analyzed by fluorescence imaging, ELISA, and Western blotting under pharmacological modulation of heme synthesis and NOX2 activity.

**Results:**

We found that SLC25A28 deficiency reduced spinal cord edema, blood‐spinal cord barrier disruption, and motor deficits. Mechanistically, SLC25A28 knockout suppressed mitochondrial iron accumulation, inhibited heme synthesis, and reduced NOX2‐mediated oxidative stress. However, SLC25A28 overexpression enhanced mitochondrial iron overload and NOX2‐driven inflammation, which could be reversed by pharmacological blockade of NOX2 or heme synthesis. Restoration of heme synthesis in A28‐MGKO microglia attenuated the anti‐inflammatory effects of SLC25A28 knockout.

**Conclusion:**

These findings demonstrate that microglial SLC25A28 regulates neuroinflammation and functional recovery after SCI by promoting mitochondrial iron‐dependent heme synthesis and NOX2 activation. Targeting the SLC25A28–heme–NOX2 axis may provide a novel therapeutic approach for SCI.

## Graphical abstract

Microglial SLC25A28 knockout reduces spinal cord injury by inhibiting mitochondrial iron accumulation and heme synthesis, leading to decreased NOX2 activation and neuroinflammation. This study reveals the SLC25A28‐heme‐NOX2 axis as a key regulator of microglial‐driven secondary damage after SCI.

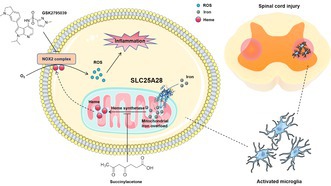

**Abbreviations**
4‐OHT: 4‐hydroxytamoxifen
5‐ALA: 5‐aminolevulinic acid
ABC: avidin‐biotin complex
ALAS1: 5‐aminolevulinate synthase 1
BMS: Basso Mouse Scale
Caspase‐1: cysteine‐aspartic protease 1
CNS: central nervous system
CRE: causing recombination enzyme (Cre recombinase)
Cx3cr1: chemokine (C‐X3‐C motif) receptor 1
CYBB: cytochrome b‐245 beta chain (gene symbol for NOX2)
DAB: 3,3′‐diaminobenzidine
DCFH‐DA: 2',7'‐dichlorofluorescin diacetate
DMEM: Dulbecco's Modified Eagle's Medium
ETC: electron transport chain
FBS: fetal bovine serum
FECH: ferrochelatase
GO: Gene Ontology
GSK2795039: NADPH oxidase 2 (NOX2) inhibitor
HE: hematoxylin–eosin
Iba1: ionized calcium binding adaptor molecule 1
IHC: immunohistochemistry
IL‐1β: interleukin‐1 beta
iNOS: inducible nitric oxide synthase
KEGG: Kyoto encyclopedia of genes and genomes
LPS: lipopolysaccharide
MGKO: microglia knockout
NF‐κB: nuclear factor kappa‐light‐chain‐enhancer of activated B cells
NLRP3: NOD‐, LRR‐ and pyrin domain‐containing protein 3
NOX2: NADPH oxidase 2
OCT: optimal cutting temperature compound
PBS: phosphate‐buffered saline
PEI: polyethylenimine
PFA: paraformaldehyde
PVDF: polyvinylidene fluoride
qPCR: quantitative polymerase chain reaction
ROS: reactive oxygen species
SA: succinylacetone
SCI: spinal cord injury
scRNA‐seq: single‐cell RNA sequencing
SDS‐PAGE: sodium dodecyl sulfate polyacrylamide gel electrophoresis
SLC25A28: solute carrier family 25 member 28
SLC25A37: solute carrier family 25 member 37
SPF: specific pathogen‐free
TEM: transmission electron microscopy
TNF‐α: tumor necrosis factor alpha
UMAP: uniform manifold approximation and projection

## 1 Introduction

Spinal cord injury (SCI) elicits a robust neuroinflammatory response, with microglia serving as the principal immune cells in the central nervous system (CNS) [1]. While microglial activation is essential for tissue repair, excessive or prolonged activation exacerbates secondary damage by amplifying inflammation and oxidative stress, ultimately hindering functional recovery [2]. Despite extensive research, the molecular mechanisms driving microglial dysregulation after SCI remain poorly understood [3].

Microglial activation is intrinsically linked to mitochondrial iron homeostasis, which plays a crucial role in regulating their inflammatory and metabolic states [4]. Upon activation, microglia increase iron uptake to meet the elevated metabolic demands. However, excessive mitochondrial iron accumulation can disrupt the function of the electron transport chain (ETC) and promote the overproduction of reactive oxygen species (ROS) through Fenton chemistry [5]. This iron‐induced ROS surge amplifies pro‐inflammatory signaling via NF‐κB and NLRP3 inflammasome activation, thereby reinforcing neurotoxic microglial polarization [6, 7]. Simultaneously, iron overload impairs mitochondrial respiration, driving a shift toward glycolytic metabolism—a state associated with sustained inflammatory responses [8]. Crucially, the regulation of mitochondrial iron balance is mediated by specialized import mechanisms, and dysregulation of these pathways has been linked to neuroinflammatory disorders [9].

Recent studies have highlighted mitochondrial iron transporters as critical regulators of microglial function in neuroinflammatory conditions [10]. The mitochondrial iron transporter SLC25A28 has been shown to mediate iron transport into mitochondria, where it plays a key role in heme biosynthesis and cellular metabolism [11, 12, 13]. In our previous study using a mouse model of intracerebral hemorrhage, we observed that microglia‐specific deletion of SLC25A28 significantly reduced neuroinflammation and improved histological outcomes, underscoring its crucial role in modulating microglial responses to central nervous system (CNS) injury [10]. These findings position SLC25A28 as a potential regulator of microglial activation in various neuropathological conditions.

In this study, we investigate the role of SLC25A28 in neuroinflammation following spinal cord injury (SCI). We hypothesize that SLC25A28 promotes microglial activation by disrupting mitochondrial iron homeostasis, thereby exacerbating oxidative stress and inflammatory damage. Through the use of microglia‐specific SLC25A28 knockout (A28‐MGKO) mice and in vitro assays, we propose a SLC25A28–heme–NOX2 regulatory axis involved in microglial‐driven neuroinflammation. Our results suggest that SLC25A28 may serve as a potential therapeutic target to promote recovery following SCI.

## 2 Materials and Methods

### 2.1 Animals

Female adult C57BL/6 mice (8–10 weeks old, 18–25 g) were obtained from the Animal Center of the Fourth Military Medical University, Xi'an, China. All animals were housed in specific pathogen‐free (SPF) conditions and provided with standard food and water ad libitum. *SLC25A28* ^*flox/flo*x^ mice and *Cx3cr1* ^*CreERT2*^ mice were sourced from Cyagen Biosciences (Suzhou, China). To generate mice with microglia‐specific deletion of SLC25A28 (*SLC25A28* ^*MGKO*^ mice, genotype: *SLC25A28* ^*flox/flox*^; *Cx3cr1‐CreERT2*), *Cx3cr1* ^*CreERT2*^ mice, which express tamoxifen‐inducible Cre recombinase, were crossed with *SLC25A28* ^*flox/flox*^ mice carrying conditional alleles of SLC25A28. Tamoxifen (75 mg/kg body weight, Sigma‐Aldrich, T5648, St. Louis, MO, USA) or corn oil (Aladdin, C116023, Shanghai, China) was administered via intraperitoneal injection once daily for 5 days. All experimental procedures were conducted 4 weeks after tamoxifen treatment. This study adhered to the ARRIVE (Animal Research: Reporting In Vivo Experiments) guidelines, and all experimental protocols were approved and overseen by the Biomedical Ethics Committee of the Health Science Center of Xi'an Jiaotong University (No. XJTUAE 2023‐2166). All the animals were randomly assigned to each experimental group.

### 2.2 Mouse SCI Model

As previously described, the SCI model was established in mice through clip compression of the exposed spinal cord at the T9 level [14]. Briefly, mice were placed under general anesthesia using 2% isoflurane in oxygen‐enriched air. A laminectomy was performed at the T9 vertebra to expose the spinal cord. The spinal cord was then compressed for 3 s using No. 5 Dumont forceps (Super Fine, Straight; 11,252–00, Fine Science Tools) while the mice were positioned in a stereotaxic apparatus to ensure consistent alignment. Although direct measurement of the applied force in grams or Newtons is not feasible with this model, consistency was ensured by using the same type of forceps, standardized hand positioning, and performing all procedures by the same experienced investigator. For the sham group, only the laminectomy was performed without subsequent compression. Postoperative care included daily monitoring, bladder management, and ensuring proper wound healing to avoid complications. These measures were taken to minimize variability and enhance the reproducibility of the model across animals.

### 2.3 Behavioral Analysis

The Basso Mouse Scale (BMS) was utilized to assess the motor function of the hind limbs in mice during open field testing [15]. Mice were evaluated weekly both before and after the injury by the same team of trained observers. These evaluations were conducted under double‐blind conditions to maintain objectivity and consistency in the experimental results.

### 2.4 Tissue Processing

After excessive inhalation of isoflurane, the mice were transcardially perfused with 0.1 M phosphate‐buffered saline (PBS, pH 7.4) followed by 4% paraformaldehyde (PFA) in PBS. The spinal cords were carefully removed, and a 1 cm segment centered on the lesion core was isolated. This segment was post‐fixed in 4% paraformaldehyde (PFA) at 4°C for 24 h, then transferred to a 30% sucrose solution at 4°C until the tissue sank, indicating complete cryoprotection. The cryoprotected spinal cord segments were rapidly encapsulated in OCT compound on dry ice to preserve the tissue structure. The frozen spinal cord segments were then sectioned at a thickness of 15 μm using a cryostat and mounted on charged glass slides.

### 2.5 Staining Procedures

#### 2.5.1 Immunohistochemistry (IHC) Staining

After sectioning, the spinal cord tissue slides were air‐dried and subjected to immunohistochemistry to detect specific antigens. The sections were first blocked with 10% normal goat serum for 1 h at room temperature to prevent non‐specific binding. Endogenous peroxidase activity was quenched by incubating the sections with 3% hydrogen peroxide for 10 min. The sections were then incubated overnight at 4°C with the primary rabbit anti‐Iba1 antibody (Abcam, ab178846; Cambridge, UK; 1 : 500) to label microglia. After washing three times with PBS, the sections were incubated with a biotinylated secondary antibody for 1 h at 37°C. The avidin‐biotin‐peroxidase complex (ABC) method was employed, and diaminobenzidine (DAB) was used as the chromogen to visualize the peroxidase activity. Finally, the sections were counterstained with hematoxylin, dehydrated, cleared in xylene, and mounted with coverslips for microscopic examination.

#### 2.5.2 Hematoxylin–Eosin (HE) Staining

The spinal cord tissue sections from the lesion core were stained with hematoxylin–eosin (HE) to evaluate the histological structure. The frozen sections were fixed in cold acetone for 10 min, followed by staining with hematoxylin solution for 5 min. After rinsing with running tap water and differentiation in 1% acid alcohol, the sections were blued in 0.2% ammonia water. They were then counterstained with eosin for 2 min, dehydrated through graded ethanol, cleared in xylene for 5 min, and mounted with neutral balsam. The stained sections were photographed using CellSens entry (Olympus, Tokyo, Japan) for further analysis.

#### 2.5.3 Nissl Staining

For Nissl staining, the frozen sections were stained with toluidine blue solution at 50°C for 40 min after rehydration in PBS. The sections were then rinsed with distilled water, dehydrated through graded concentrations of ethanol (70%, 80%, 95%, and 100%) for 3 min each, cleared in xylene for 5 min, and mounted with neutral balsam. The number of surviving neurons was quantified using ImageJ software (National Institutes of Health, Bethesda, MD, USA).

#### 2.5.4 Transmission Electron Microscopy (TEM)

Following transcardial perfusion with 0.1 M phosphate‐buffered saline (PBS, pH 7.4), spinal cord tissues were fixed by immersion in 2.5% glutaraldehyde in PBS for 2 h at 4°C. After fixation, tissues were washed three times in PBS for 10 min each. Post‐fixation was performed with 1% osmium tetroxide (OsO_4_) in PBS for 2 h on ice. The tissues were then dehydrated through a graded ethanol series, followed by infiltration with a 1:1 mixture of propylene oxide and epoxy resin. The samples were embedded in fresh epoxy resin and polymerized at 60°C for 48 h. Thin sections (70–90 nm) were cut using an ultramicrotome, stained with uranyl acetate and lead citrate, and examined under a transmission electron microscope (TEM) at an appropriate magnification (10,000×–50,000×) to assess the ultrastructure of neurons and microglia.

### 2.6 Blood‐Spinal Cord Barrier Permeability

The permeability of the blood‐spinal cord barrier was assessed using Evans blue dye. Mice (*n* = 3 per group) were injected with Evans blue dye (2%, 4 mL/kg, Aladdin, Shanghai, China) through the tail vein 3 days after spinal cord injury (SCI). Two hours post‐injection, the mice were anesthetized using isoflurane gas anesthesia, followed by transcardial perfusion with PBS to remove intravascular Evans blue. After perfusion, the entire spinal cord, including the brain, was carefully removed for gross observation and photography to document the distribution of Evans blue dye, providing an overview of the blood‐spinal cord and blood–brain barrier integrity. A 1 cm segment centered on the lesion core was then isolated from the spinal cord for further analysis. This segment was rapidly encapsulated in optimal cutting temperature (OCT) compound on dry ice to preserve the tissue structure. The tissue was subsequently homogenized in dimethylformamide solution (1 mL/100 mg tissue) and incubated at 60°C for 24 h. After centrifugation for 5 min, the supernatants were collected, and the absorbance was measured using a spectrophotometer at 620 nm. The results were expressed as the amount of Evans blue leakage per gram of spinal cord tissue protein.

### 2.7 Spinal Cord Edema Assessment

To evaluate spinal cord edema, mice (*n* = 3 per group) were sacrificed 3 days post‐spinal cord injury (SCI) following anesthesia with isoflurane. The entire spinal cord was carefully extracted, and a 1 cm segment centered on the lesion core was isolated. This segment was immediately weighed to obtain the wet weight. The tissue was then dried at 100°C for 72 h to achieve a constant weight, which was recorded as the dry weight. Spinal cord water content was calculated using the formula:

$$\left(\mathrm{Wet}\;\text{weight}-\mathrm{Dry}\;\text{weight}\right)/\mathrm{Wet}\;\text{weight}\times 100\%.$$

### 2.8 Cell Culture and Treatment

Murine BV2 microglial cells (Cell Bank of the Chinese Academy of Sciences, Shanghai, China) and HEK293T human embryonic kidney cells (ATCC CRL‐3216, Manassas, VA, USA) were cultured in Dulbecco's Modified Eagle's Medium (DMEM, Thermo Fisher Scientific, Waltham, MA, USA), supplemented with 10% fetal bovine serum (FBS, ExCell Bio, Shanghai, China) and a penicillin/streptomycin antibiotic mix. All cell cultures were maintained in a humidified incubator at 37°C with 5% CO_2_. Stable SLC25A28 overexpressing cell lines were established using lentiviral transduction, with cells transfected with either the SLC25A28 overexpression construct or an empty vector control (pLenti‐C‐Myc‐DDK‐IRES‐Puro). The lentiviral particles were produced by co‐transfecting HEK293T cells with the lentiviral vector and packaging plasmids pVSVG and pAX2, using polyethylenimine (PEI). After an overnight incubation, the viral supernatant was harvested at 72 h, filtered through a 0.45 μm filter, and used to infect BV2 cells in the presence of polybrene (4 mg/mL). Infected cells were then selected with puromycin (2 μg/mL).

Primary microglia were isolated from female *SLC25A28* ^*MGKO*^ neonatal mice (postnatal Days 1–3). After removal of meninges, the brains were minced and digested with 0.125% trypsin and 0.2 mg/mL DNase I, followed by centrifugation at 1500 rpm for 3 min. Mixed glial cultures were maintained for 14 days, then shaken at 200 rpm for 6 h at 37°C to detach microglia. Collected microglia were plated into fresh flasks. For gene deletion, cells were treated with 1 μM 4‐hydroxytamoxifen (4‐OHT, Sigma‐Aldrich, H7904, St. Louis, MO, USA) for 4 days.

For in vitro spinal cord injury (SCI) modeling, primary microglia were stimulated with lipopolysaccharide (LPS, *E. coli* O111:B4, Sigma‐Aldrich, L4391, St. Louis, MO, USA) at concentrations of 0.5, 1, or 2 μg/mL for 24 h in complete DMEM. A concentration of 1 μg/mL was selected for all subsequent experiments because it induced a robust and consistent activation of microglia (as evidenced by morphological changes and pro‐inflammatory marker expression; see Figure 2a), which is a well‐established model for neuroinflammatory stimulation and aligns with concentrations widely used in the field [16].

To investigate the regulatory mechanisms of microglial activation, pharmacological interventions were employed. Specifically, the NOX2 inhibitor GSK2795039 (10 μM, MedChemExpress, HY‐18950, Monmouth Junction, NJ, USA) was added 1 h prior to LPS stimulation in SLC25A28‐overexpressing BV2 cells to suppress NOX2 activity. To inhibit heme biosynthesis, succinylacetone (SA; 100 μM, Sigma‐Aldrich, 20011, St. Louis, MO, USA), a competitive inhibitor of porphobilinogen synthase that blocks the conversion of δ‐aminolevulinic acid to porphobilinogen, was applied 2 h before LPS treatment in the same overexpression model. Conversely, in SLC25A28 knockout primary microglia, 5‐aminolevulinic acid (5‐ALA; 100 μM, Sigma‐Aldrich, A7793, BioReagent, ≥ 98%, St. Louis, MO, USA), a precursor in heme biosynthesis that bypasses the ALAS1‐dependent rate‐limiting step, was administered for 24 h to restore heme synthesis.

### 2.9 Cell Morphology Imaging

To evaluate cell volume and activation status, cells were imaged directly under a bright‐field microscope (Olympus, IX‐83, Tokyo, Japan). Cells were cultured in 12‐well plates at a density of 1 × 10^5^ cells per well. After treatments, cells were examined for morphological changes indicative of activation.

The images were captured at 10× or 20× magnification. The cell area and morphology were analyzed using ImageJ software (National Institutes of Health, Bethesda, MD, USA). The body area (cell size) was measured by tracing the perimeter of individual cells, and the results were expressed as μm^2^. The fold change in the body area was calculated by normalizing the cell area of treated cells to that of the control group. Activated cells were identified by morphological features such as increased cell body size and changes in cytoskeletal structure, including larger cell body size and irregular shape.

### 2.10 Mitochondrial Isolation and Iron Assay

Mitochondria were isolated from primary microglial cells and spinal cord tissue using mitochondria isolation kits (Beyotime Biotechnology, C3601 and C3606, Shanghai, China). Briefly, microglia or spinal cord tissue were homogenized in mitochondria isolation solution containing PMSF and incubated on ice for 15 min. A glass homogenizer was used to grind the samples, followed by centrifugation at 1000 × g for 10 min at 4°C. The supernatant was transferred to a new tube and centrifuged again at 3500 × g for 10 min. The pellet containing mitochondria was collected.

To measure mitochondrial iron content, a colorimetric ferrozine‐based assay was used. Mitochondrial pellets were incubated with 50 mM NaOH for 2 h at room temperature. Iron was then extracted by adding 10 mM HCl and iron‐releasing buffer (1.4 M HCl with 4.5% KMnO_4_, 1:1), followed by incubation at 60°C for 2 h. After incubation, 30 μL of detection buffer (6.5 mM ferrozine, 6.5 mM neocuproine, 2.5 M ammonium acetate, and 1 M ascorbic acid) was added and incubated for 30 min at room temperature. Absorbance was measured at 550 nm, and the iron content was calculated using FeCl_3_ standard curves, expressed as nmol of iron per milligram of protein.

### 2.11 RNA Extraction and qRT‐PCR

Total RNA was extracted from tissue or cells using TRIzol reagent (Takara Bio Inc., Kusatsu, Shiga, Japan) according to the manufacturer's instructions. RNA was then reverse transcribed into cDNA using PrimeScript RT Master Mix and quantified with the SYBR Green PCR kit (both from Takara Bio Inc.). Gene expression levels were normalized to 18S rRNA, and the relative expression of target genes was calculated using the comparative threshold cycle method ($2^{-\Delta \Delta C_{t}}$). The primers used for PCR are listed in Table S1.

### 2.12 Mito‐FerroGreen Staining

Mitochondrial FerroGreen (Mito‐FerroGreen, Dojindo, Kumamoto, Japan) was used to assess the level of Fe^2+^ in mitochondria. Cells were seeded in a 12‐well plate at a density of 1 × 10^5^ cells per well. The Mito‐FerroGreen solution (5 μM) was added to the cells and incubated for 30 min. After incubation, the cells were washed three times with serum‐free medium. To induce mitochondrial iron accumulation, cells were then incubated with ferric citrate (100 μM) for 1 h. Fluorescent signals were observed using a fluorescence microscope.

### 2.13 ROS Detection

ROS levels were measured using the ROS detection assay kit (Beyotime, S0033S, Shanghai, China) following the manufacturer's protocol. Briefly, cells were incubated with 2′,7′‐dichlorofluorescin diacetate (DCFH‐DA) for 30 min at 37°C. After staining, the cells were washed three times with serum‐free medium. Fluorescence was then captured using a fluorescence microscope (Olympus IX‐83, Tokyo, Japan).

### 2.14 Western Blot Analysis

Proteins were separated by 10% SDS‐PAGE and transferred to polyvinylidene fluoride (PVDF) membranes (Millipore, Burlington, MA, USA). The membranes were incubated overnight at 4°C with primary antibodies: rabbit anti‐SLC25A28 polyclonal antibody (1:1000, Invitrogen, PA5‐31839, Carlsbad, CA, USA), rabbit anti‐SLC25A37 polyclonal antibody (1:500, Invitrogen, PA5‐119913, Carlsbad, CA, USA), rabbit anti‐NOX2 monoclonal antibody (1:1000, Abmart, TD6520, Shanghai, China), rabbit anti‐iNOS polyclonal antibody (1:200, Abcam, ab15323, Cambridge, UK), rabbit anti‐total and anti‐cleaved Caspase‐1 polyclonal antibody (1:1000, Abmart, P79884R2, Shanghai, China), rabbit anti‐ALAS1 polyclonal antibody (1:1000, Abmart, PS04581, Shanghai, China), and rabbit anti‐FECH polyclonal antibody (1:1000, Abmart, PS07310, Shanghai, China). After washing the membranes three times, the membranes were incubated with secondary antibodies for 1 h at room temperature. Protein bands were visualized using ECL detection reagents (Thermo Fisher Scientific, Waltham, MA, USA). Imaging was performed using a ChemiDoc imaging system (Bio‐Rad Laboratories, Hercules, CA, USA), and protein expression levels were quantified by densitometry. The results were normalized to β‐actin as the loading control.

### 2.15 Enzyme‐Linked Immunosorbent Assay (ELISA)

ELISA was performed according to the manufacturer's instructions (A&E Bio, Shaanxi, China) to assess the concentrations of IL‐1β (Catalog No. AE20220) and TNF‐α (Catalog No. AE20533). The optical density of each well at 450 nm was determined using a microplate reader. All experiments were performed in triplicate.

### 2.16 Single‐Cell RNA Sequencing Data Analysis

Single‐cell RNA sequencing (scRNA‐seq) data were obtained from the dataset published by Li et al. [17] (https://doi.org/10.6084/m9.figshare.17702045.v2) and analyzed using the Seurat package (version 4.0) in R. Raw sequencing data were processed with CellRanger (version 3.1.0) and aligned to the mm10 reference genome (Ensembl release 84). Low‐quality cells were excluded based on the following criteria: fewer than 200 detected genes or over 20% of transcripts derived from mitochondrial genes.

Data normalization was performed using the LogNormalize method with a scale factor of 10,000. The top 5000 most variable genes were identified and used for downstream analyses. The data were then scaled, and unsupervised clustering was conducted using the FindClusters function at a resolution of 0.8. Cell‐type identities were assigned based on established marker genes, particularly microglial markers such as Iba1 and Cx3cr1. Differential gene expression analysis was carried out using the FindMarkers function, with genes considered significant if they exhibited an adjusted *p*‐value of less than 0.05.

Pathway enrichment analysis was conducted using the Gene Ontology (GO) and Kyoto encyclopedia of genes and genomes (KEGG) databases. Enriched pathways were visualized using bubble plots. Uniform Manifold Approximation and Projection (UMAP) was applied for dimensionality reduction and visualization of clustering results, while heatmaps were used to display differentially expressed genes across clusters.

### 2.17 Statistical Analysis

All data are presented as mean ± SEM. Statistical analyses were performed using GraphPad Prism (version 8.0.2). Data were compared using Student's *t*‐test for two‐group comparisons, or ANOVA followed by *t*‐tests for pairwise comparisons among multiple groups. A *p*‐value of less than 0.05 was considered statistically significant.

## 3 Results

### 3.1 The Mitochondrial Iron Transporter SLC25A28 Is Closely Related to Microglial Activation in Spinal Cord Injury

**FIGURE 1:**
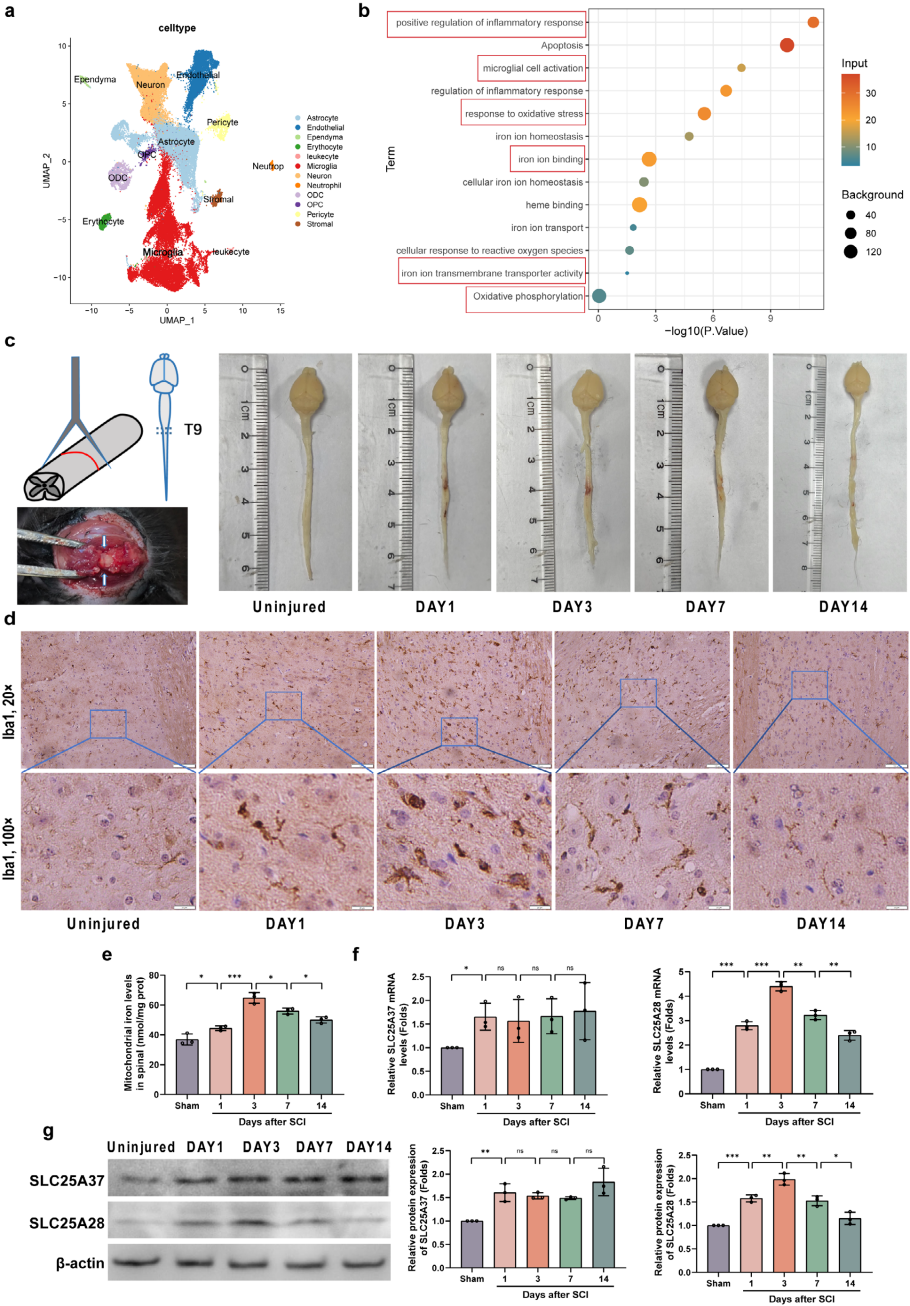
Temporal analysis of microglial activation and mitochondrial iron content following spinal cord injury (SCI). (a) UMAP plot of single‐cell transcriptomic data from spinal cord tissue following injury, showing the predominant cell types post‐SCI. (b) GO‐KEGG enrichment analysis of differentially expressed genes in microglia post‐SCI. (c) Schematic diagram of the spinal cord clip injury model and gross observation of spinal cord tissue at different time points (1, 3, 7, and 14 days post‐injury). (d) Iba1 immunohistochemical staining of spinal cord tissue at 1, 3, 7, and 14 days post‐injury, showing microglial activation. Upper row: 20× magnification (scale bar = 50 μm); lower row: 100× magnification (scale bar = 10 μm). (e) Measurement of mitochondrial iron content in spinal cord tissue at various time points (1, 3, 7, and 14 days post‐injury) using a colorimetric ferrozine‐based assay after mitochondrial isolation from spinal cord tissue (*n* = 3). (f) qPCR analysis of SLC25A28 mRNA expression in spinal cord tissue at different time points post‐injury (*n* = 3). (g) Western blot analysis of SLC25A28 and SLC25A37 expression in spinal cord tissue at 1, 3, 7, and 14 days post‐injury (*n* = 3). *p < 0.05, **p < 0.01, ***p < 0.001.

Previous studies have highlighted the crucial role of microglial activation in spinal cord injury (SCI), where microglia‐mediated inflammation exacerbates secondary spinal cord damage. To further investigate microglial involvement, we first analyzed publicly available single‐cell transcriptomic data. Our results confirmed that microglia are the predominant cell type post‐SCI (Figure 1a), with activation progressively increasing over time. Specifically, microglial activation peaked on Days 3 and 7 post‐injury, followed by a decline (Figure S1). Pathway enrichment analysis revealed significant upregulation in pathways associated with microglial activation, oxidative stress, inflammatory responses, ROS production, mitochondrial iron metabolism, and mitochondrial dysfunction (Figure 1b). Given the strong link between microglial‐mediated inflammation and iron metabolism, we focused on mitochondrial iron homeostasis as a key mediator of microglial activation during SCI.

To investigate further, we used a mouse spinal cord clip injury model and assessed tissue changes at various time points (uninjured, 1, 3, 7, and 14 days post‐injury) through gross observation (Figure 1c) and Iba1 immunohistochemistry (Figure 1d). The results revealed that spinal cord injury and microglial activation peaked on Day 3. Our prior research demonstrated that the knockout of the mitochondrial iron transporter SLC25A28 alleviates microglial activation and improves outcomes in a mouse model of intracerebral hemorrhage [10]. Based on its involvement in microglial activation and mitochondrial iron transport, we hypothesized that SLC25A28 may also play a significant role in SCI. To evaluate this hypothesis, we first assessed mitochondrial iron content in injured spinal cord tissue. We found that mitochondrial iron levels peaked on Day 3 post‐injury, coinciding with the peak of microglial activation and tissue injury (Figure 1e). Although SLC25A37, another mitochondrial iron transporter, shares some structural and functional similarities with SLC25A28, its expression did not follow the same temporal pattern as SLC25A28. SLC25A37 expression increased post‐injury, but it did not correlate with the peak of microglial activation or injury (Figure 1f,g). These results led us to focus on SLC25A28 as the primary target for further investigation, given its temporal correlation with microglial activation and its more direct involvement in SCI.

**FIGURE 2:**
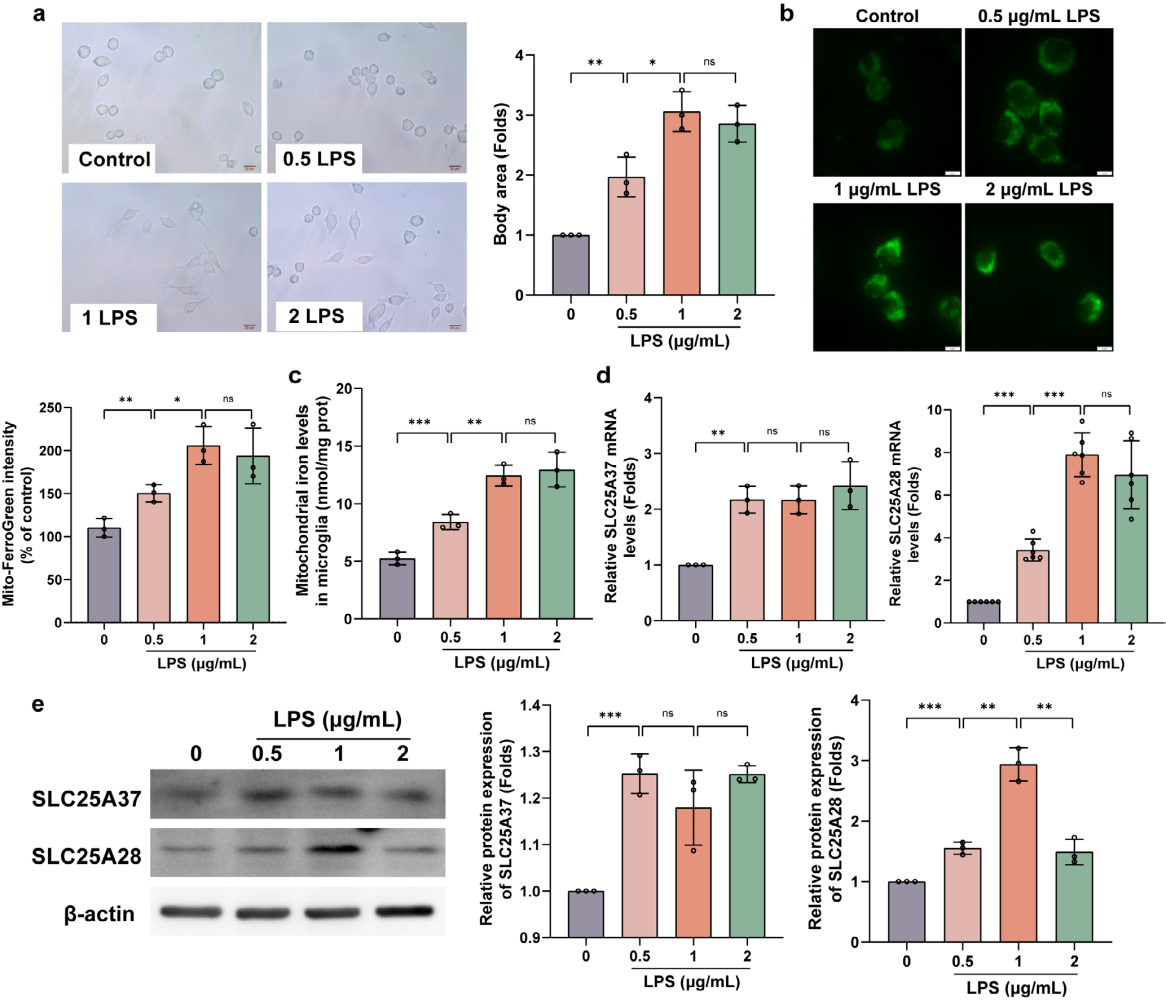
In vitro microglial activation and mitochondrial iron content after LPS treatment. LPS (0.5, 1, and 2 μg/mL) for 24 h. (a) Cell morphology of primary microglial cells after LPS (0.5, 1, and 2 μg/mL) treatment for 24 h, assessed by body area (Scale bar = 20 μm, *n* = 3). (b) Mitochondrial iron content measured in microglial cells using a ferrozine‐based assay following mitochondrial isolation (*n* = 3). (**c**) Mito‐FerroGreen staining showing mitochondrial iron content in primary microglial cells (Scale bar = 5 μm, *n* = 3). (d) qPCR analysis of SLC25A28 and SLC25A37 mRNA expression in primary microglial cells (*n* = 3). (e) Western blot analysis of SLC25A28 and SLC25A37 expression in primary microglial cells (*n* = 3). Data are expressed as mean ± SEM. *p < 0.05, **p < 0.01, ***p < 0.001.

We next established an in vitro SCI model by treating primary microglial cells with lipopolysaccharide (LPS) to simulate SCI‐like conditions. Treatment with 0.5, 1, and 2 μg/mL LPS for 24 h revealed that microglial activation was most pronounced at 1 μg/mL, with no further activation observed at 2 μg/mL (Figure 2a). Mitochondrial iron assays (Figure 2b) and Mito‐FerroGreen staining (Figure 2c) confirmed that mitochondrial iron levels reached a threshold at 1 μg/mL LPS. Furthermore, qPCR and Western blot analyses showed that SLC25A28 expression peaked at 1 μg/mL LPS in a dose‐dependent manner (Figure 2d,e). Unlike SLC25A37, whose expression also increased but did not correlate with the peak activation, SLC25A28 displayed a clear association with microglial activation and mitochondrial iron accumulation. These findings highlight the potential role of SLC25A28 in regulating microglial activation after SCI, particularly through its influence on mitochondrial iron transport and inflammatory responses.

### 3.2 Microglia‐Specific SLC25A28 Knockout Alleviates SCI in Mice

**FIGURE 3:**
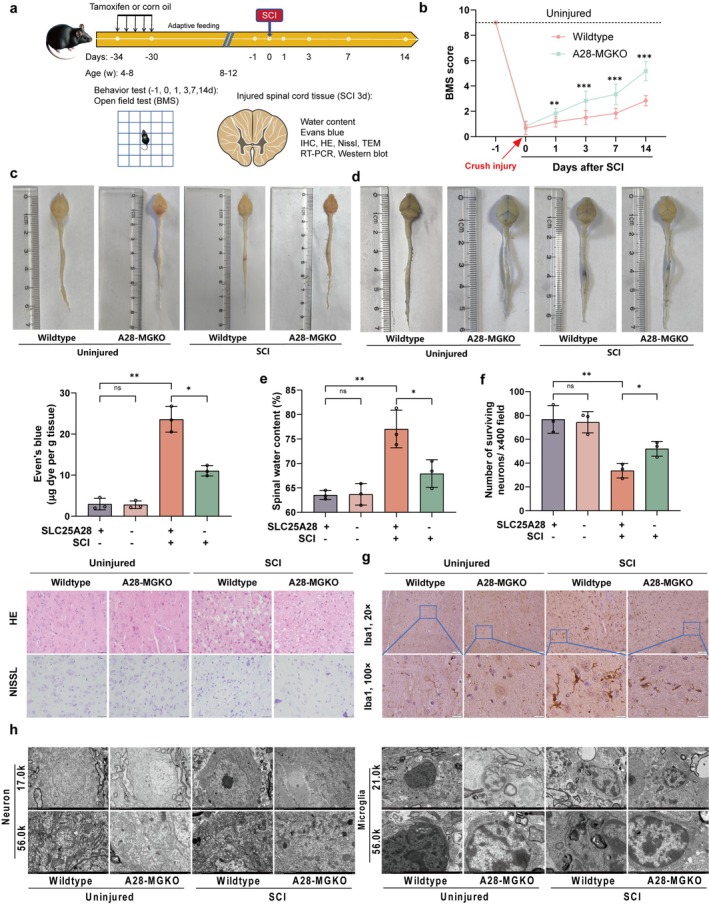
Microglia‐specific SLC25A28 knockout alleviates spinal cord injury (SCI) in mice. (a) Experimental timeline illustrating tamoxifen‐induced generation of A28‐MGKO mice and analyses performed at Day 3 post‐SCI. (b) Basso Mouse Scale (BMS) scores for locomotor function at Day 3 post‐SCI (*n* = 6). (c) Representative gross images of spinal cords showing lesion severity at Day 3 post‐SCI. (d) Evans blue staining and quantification of blood‐spinal cord barrier permeability (*n* = 5). (e) Spinal cord water content measurement to assess edema (*n* = 3). (f) Hematoxylin and eosin (HE) and Nissl staining showing tissue preservation and neuronal survival at Day 3 post‐SCI (*n* = 3). Scale bar = 50 μm. (g) Iba1 immunohistochemical staining showing microglial activation. Upper row: 20× magnification (scale bar = 50 μm); lower row: 100× magnification (scale bar = 10 μm). (h) Transmission electron microscopy (TEM) images of spinal cord neurons and microglia at Day 3 post‐SCI, showing neuronal morphology at 17 k and 56 k magnification and microglial activation at 21 k and 56 k magnification. Scale bar = 5 μm for 17 k and 21 k magnification, 1 μm for 56 k magnification. Data are expressed as mean ± SEM. *p < 0.05, **p < 0.01, ***p < 0.001.

To explore the role of SLC25A28 in microglial activation and inflammation following spinal cord injury (SCI), we generated tamoxifen‐induced microglia‐specific SLC25A28 knockout (A28‐MGKO) mice (see Figure S2). Behavioral changes were monitored at various time points, and histological and biochemical markers were assessed on Day 3 post‐SCI (Figure 3a). Behavioral changes were evaluated using the Basso Mouse Scale (BMS), which showed a significant improvement in neurological function scores (normal score 9, maximal deficit 0) in A28‐MGKO mice compared to the control group (Figure 3b). Gross spinal cord images at Day 3 post‐SCI revealed significantly less damage in A28‐MGKO mice compared to controls (Figure 3c). Evans blue staining was used to assess blood‐spinal cord barrier permeability, and gross images and quantification (Figure 3d) indicated improved barrier integrity in A28‐MGKO mice. Measurement of spinal cord water content also showed reduced edema in A28‐MGKO mice (Figure 3e). These results suggest that microglia‐specific SLC25A28 knockout alleviates the blood‐spinal cord barrier disruption and edema following SCI.

Histological analysis further supported these findings. Hematoxylin and eosin (HE) staining (Figure 3f) demonstrated that A28‐MGKO significantly mitigated spinal cord injury. In the control group, typical injury features included extensive tissue disorganization and vacuolation, indicating structural damage. In contrast, A28‐MGKO mice exhibited better‐preserved spinal architecture with less vacuolar degeneration, suggesting improved tissue integrity. Nissl staining (Figure 3f) and its quantification showed a significant increase in surviving neurons in A28‐MGKO mice, indicating improved neuronal survival following SCI. Immunohistochemical (IHC) staining for Iba1 (Figure 3g) revealed that control mice exhibited activated microglial morphology with hypertrophic cell bodies and shortened, less branched processes after SCI, whereas A28‐MGKO mice maintained more ramified morphology, suggesting that microglia‐specific SLC25A28 knockout attenuates activation post‐SCI. Transmission electron microscopy (TEM) analysis (Figure 3h) showed significant neuronal damage in the SCI group, particularly mitochondrial swelling and cristae disruption, along with activated microglia. In contrast, A28‐MGKO mice exhibited reduced neuronal damage and microglial activation, with microglia displaying a more resting morphology. These findings suggest that A28‐MGKO provides both neuronal protection and suppression of microglial activation.

In summary, these findings indicate that microglia‐specific SLC25A28 knockout reduces neuroinflammation, mitigates spinal cord injury, and improves functional recovery following SCI.

### 3.3 Microglia‐Specific SLC25A28 Knockout Reduces Mitochondrial Iron Accumulation and Neuroinflammation

**FIGURE 4:**
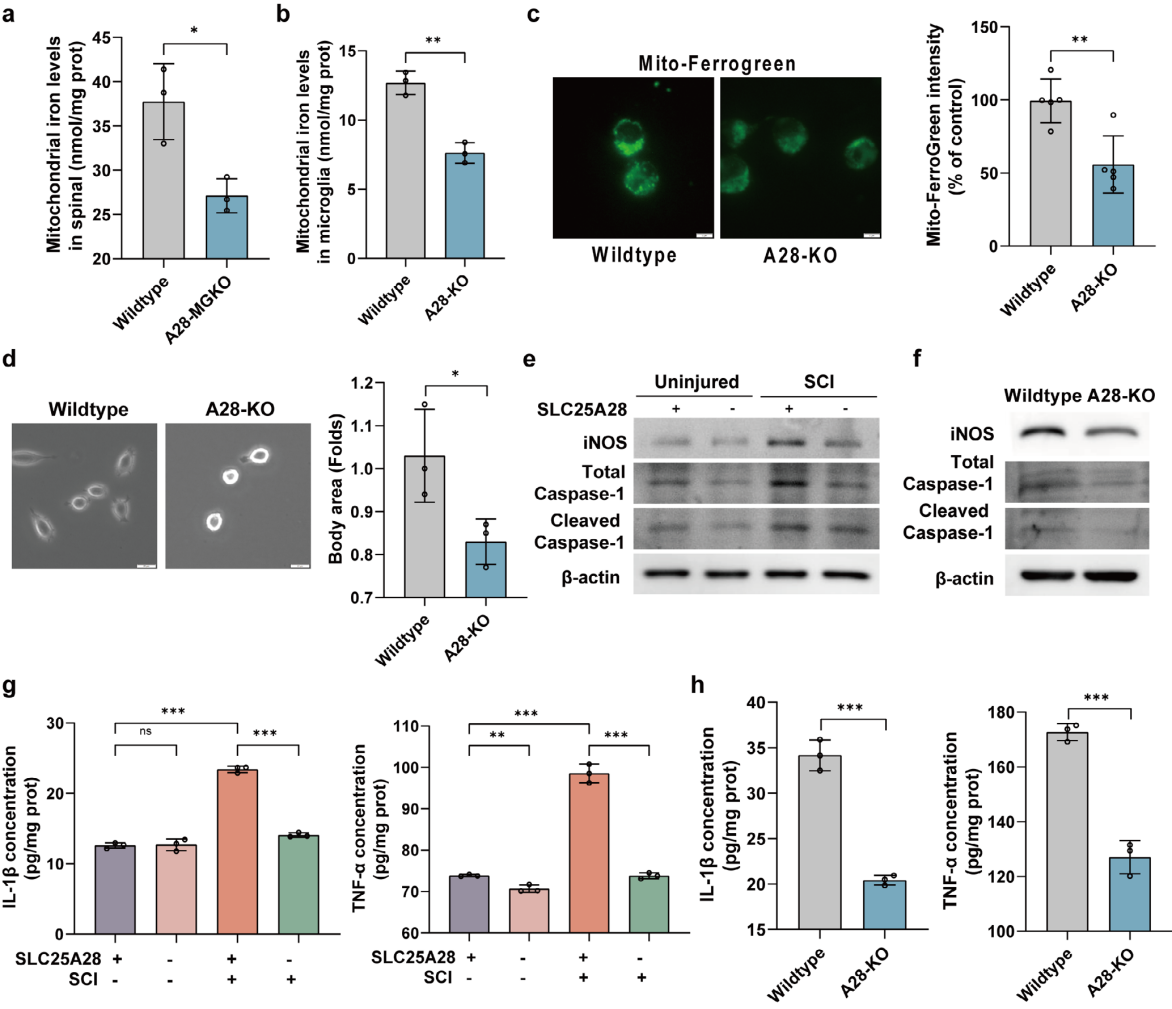
Microglia‐specific SLC25A28 knockout reduces mitochondrial iron content and attenuates neuroinflammation. (a) Mitochondrial iron content in spinal cord tissues from A28‐MGKO and control mice measured by ferrozine‐based assay (*n* = 3). (b) Mitochondrial iron levels in primary microglia with SLC25A28 knockout (A28‐KO) and control cells (*n* = 3). (c) Mito‐FerroGreen staining showing mitochondrial iron content in A28‐KO and control microglia (Scale bar = 5 μm, *n* = 5). (d) Morphological analysis of A28‐KO microglia showing reduced cell body area (Scale bar = 20 μm, *n* = 3). (e) Western blot analysis of inflammatory markers (iNOS, total caspase‐1, and cleaved caspase‐1) in spinal cord tissues (*n* = 3). (f) Western blot analysis of inflammatory markers in A28‐KO and control primary microglia (*n* = 3). (g) ELISA quantification of IL‐1β and TNF‐α in spinal cord tissues (*n* = 3). (h) ELISA analysis of IL‐1β and TNF‐α secretion from A28‐KO and control primary microglia (*n* = 3). Data are expressed as mean ± SEM. *p < 0.05, **p < 0.01, ***p < 0.001.

Based on our findings that microglia‐specific SLC25A28 knockout (A28‐MGKO) improves functional recovery after spinal cord injury (SCI), we aimed to investigate the underlying mechanisms by focusing on mitochondrial iron homeostasis and its relationship to neuroinflammation. First, we quantified mitochondrial iron levels in spinal cord tissues and primary microglia following A28‐MGKO. In isolated spinal cord mitochondria, A28‐MGKO resulted in a significant reduction in iron content compared to controls (Figure 4a). Similarly, SLC25A28 knockout in primary microglia (A28‐KO) led to reduced mitochondrial iron levels (Figure 4b). Mito‐FerroGreen staining further confirmed this reduction, showing decreased fluorescence intensity in A28‐KO microglia (Figure 4c).

Consistent with the iron quantification results, morphological analysis demonstrated that A28‐KO primary microglia exhibited smaller cell bodies and simplified branching patterns. This morphological change suggested that SLC25A28 deletion promotes microglial quiescence (Figure 4d). To determine the functional consequences of reduced mitochondrial iron, we examined the expression of key inflammatory markers. Western blot analysis of spinal cord tissues revealed that A28‐MGKO significantly downregulated key components of the neuroinflammatory cascade, including both total caspase‐1 and cleaved caspase‐1, as well as the pro‐inflammatory enzyme iNOS (Figure 4e). This anti‐inflammatory phenotype was replicated in primary microglia, where A28‐KO cells showed comparable reductions in the same inflammatory markers (Figure 4f). To further confirm the reduction in pro‐inflammatory cytokine production, we performed ELISA assays to quantify IL‐1β and TNF‐α levels in spinal cord tissues. In response to SCI, both cytokines were significantly elevated, while this increase was markedly reduced in A28‐MGKO mice (Figure 4g). Similar results were observed in primary microglia, where A28‐KO cells exhibited a substantial reduction in IL‐1β and TNF‐α secretion compared to controls (Figure 4h). The parallel findings in vivo and in vitro strongly support the conclusion that microglial SLC25A28 knockout attenuates neuroinflammation through modulation of mitochondrial iron homeostasis.

Together, these results indicate that SLC25A28 deficiency in microglia mitigates mitochondrial iron overload and suppresses neuroinflammatory responses following SCI.

### 3.4 SLC25A28 Regulates Microglial Activation Through NOX2‐Dependent ROS Production

**FIGURE 5:**
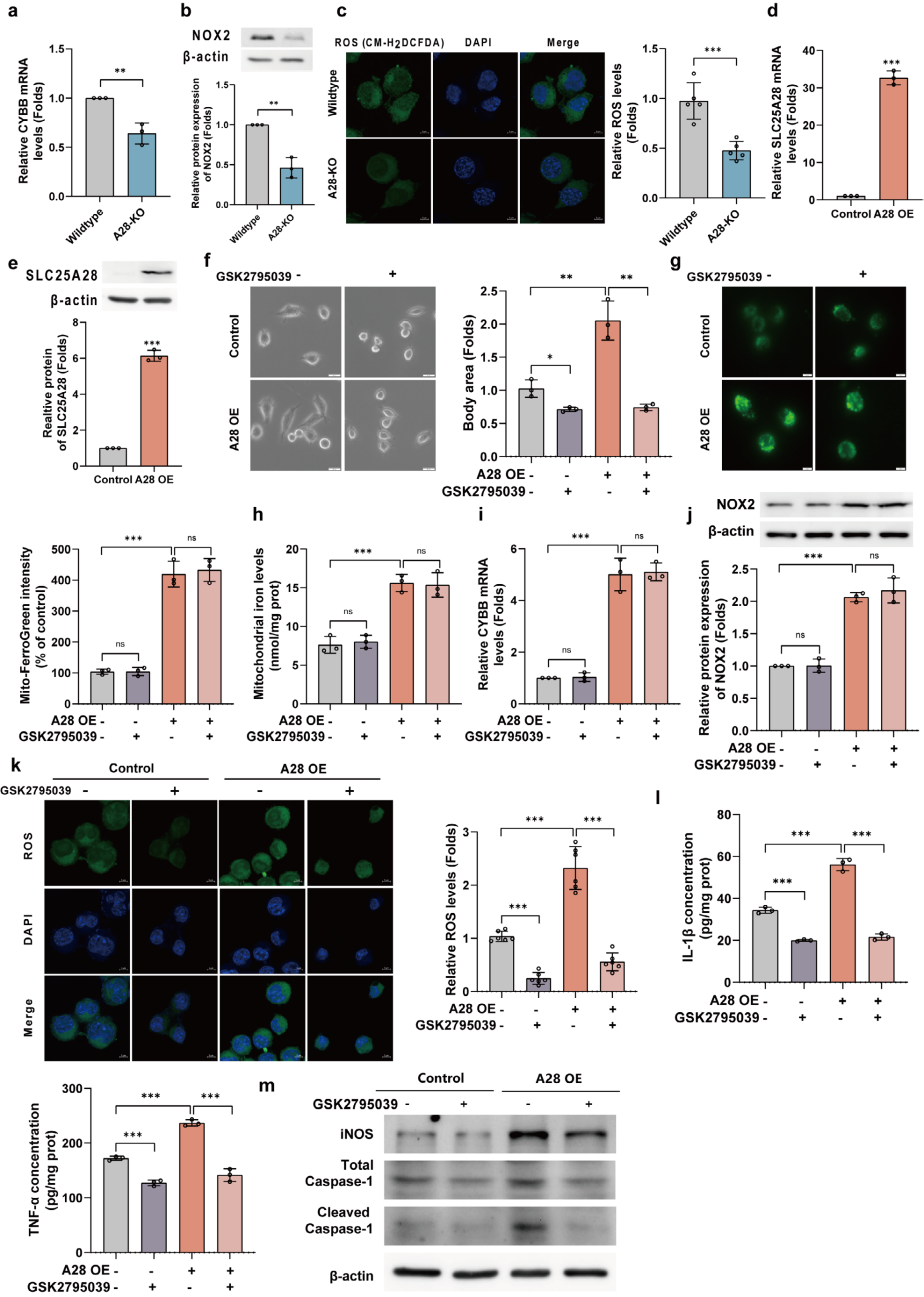
SLC25A28 regulates microglial activation via NOX2‐dependent ROS pathway. (a) CYBB mRNA levels in SLC25A28‐KO versus control microglia (qPCR, *n* = 3). (b) NOX2 protein expression in microglia (Western blot, *n* = 3). (c) Intracellular ROS levels in microglia assessed by DCFH‐DA staining (scale bar = 5 μm, *n* = 5). (d) SLC25A28 mRNA expression in overexpression (OE) versus control BV2 cells (qPCR, *n* = 3). (e) SLC25A28 protein levels in OE and control cells (Western blot, *n* = 3). (f) Microglial morphology with/without GSK2795039 (10 μM) treatment; cell body area was quantified (scale bar = 20 μm, *n* = 3). (g) Mitochondrial iron visualization using Mito‐FerroGreen staining (scale bar = 5 μm). (h) Quantification of mitochondrial iron content by ferrozine assay (*n* = 3). (i) CYBB mRNA expression in OE cells with or without GSK2795039 treatment (qPCR, *n* = 3). (j) NOX2 protein expression in OE cells with or without GSK2795039 treatment (Western blot, *n* = 3). (k) ROS levels in OE cells with or without GSK2795039 treatment, measured by DCFH‐DA staining (scale bar = 20 μm, *n* = 6). (l) Expression of inflammatory markers (iNOS, total caspase‐1, and cleaved caspase‐1) in OE cells with or without GSK2795039 treatment (Western blot, *n* = 3). (m) ELISA quantification of IL‐1β and TNF‐α in OE cells with or without GSK2795039 treatment (*n* = 3). Data are expressed as mean ± SEM. *p < 0.05, **p < 0.01, ***p < 0.001.

To investigate the role of SLC25A28 in microglial activation, we first examined the impact of SLC25A28 knockout (A28‐KO) in primary microglia. We found that A28‐KO significantly reduced NOX2 expression at both mRNA (CYBB, Figure 5a) and protein levels (Figure 5b). Correspondingly, reactive oxygen species (ROS) production, assessed by DCFH‐DA staining, was notably lower in A28‐KO microglia compared to controls (Figure 5c), indicating reduced oxidative stress.

To investigate the functional role of SLC25A28, we established SLC25A28‐overexpressing BV2 cells (A28 OE). Successful overexpression of SLC25A28 was confirmed by qPCR (Figure 5d) and Western blot (Figure 5e). In these A28 OE cells, we observed a significant increase in mitochondrial iron content, as demonstrated by Mito‐FerroGreen staining (Figure 5g) and quantitative analysis (Figure 5h). Importantly, this iron accumulation was unaffected by the NOX2 inhibitor GSK2795039, suggesting that NOX2 activation occurs downstream of mitochondrial iron accumulation. Morphological analysis of A28 OE cells revealed enlarged cell bodies, a hallmark of microglial activation. This phenotype was reversed by treatment with GSK2795039 (Figure 5f), indicating that NOX2 plays a critical role in regulating microglial morphology.

Furthermore, A28 OE cells exhibited a significant increase in NOX2 expression, which was unaffected by GSK2795039 treatment (Figure 5i,j). To assess the functional impact of NOX2 activation, we measured ROS levels and found a marked increase in A28 OE cells compared to controls (Figure 5k). This elevation was abolished by GSK2795039, indicating that ROS production was NOX2‐dependent.

We next evaluated inflammatory marker expression in A28 OE cells, including iNOS, total caspase‐1, and cleaved caspase‐1. All were significantly upregulated and normalized by NOX2 inhibition (Figure 5l). ELISA assays further confirmed increased IL‐1β and TNF‐α secretion in A28 OE cells, which were reversed by GSK2795039 (Figure 5m).

Together, these findings suggest that SLC25A28 promotes microglial activation via a NOX2‐dependent ROS pathway, with mitochondrial iron accumulation as an upstream trigger of NOX2 activation and downstream neuroinflammatory responses.

### 3.5 SLC25A28 Overexpression Promotes NOX2 Activation Through Enhanced Heme Biosynthesis

**FIGURE 6:**
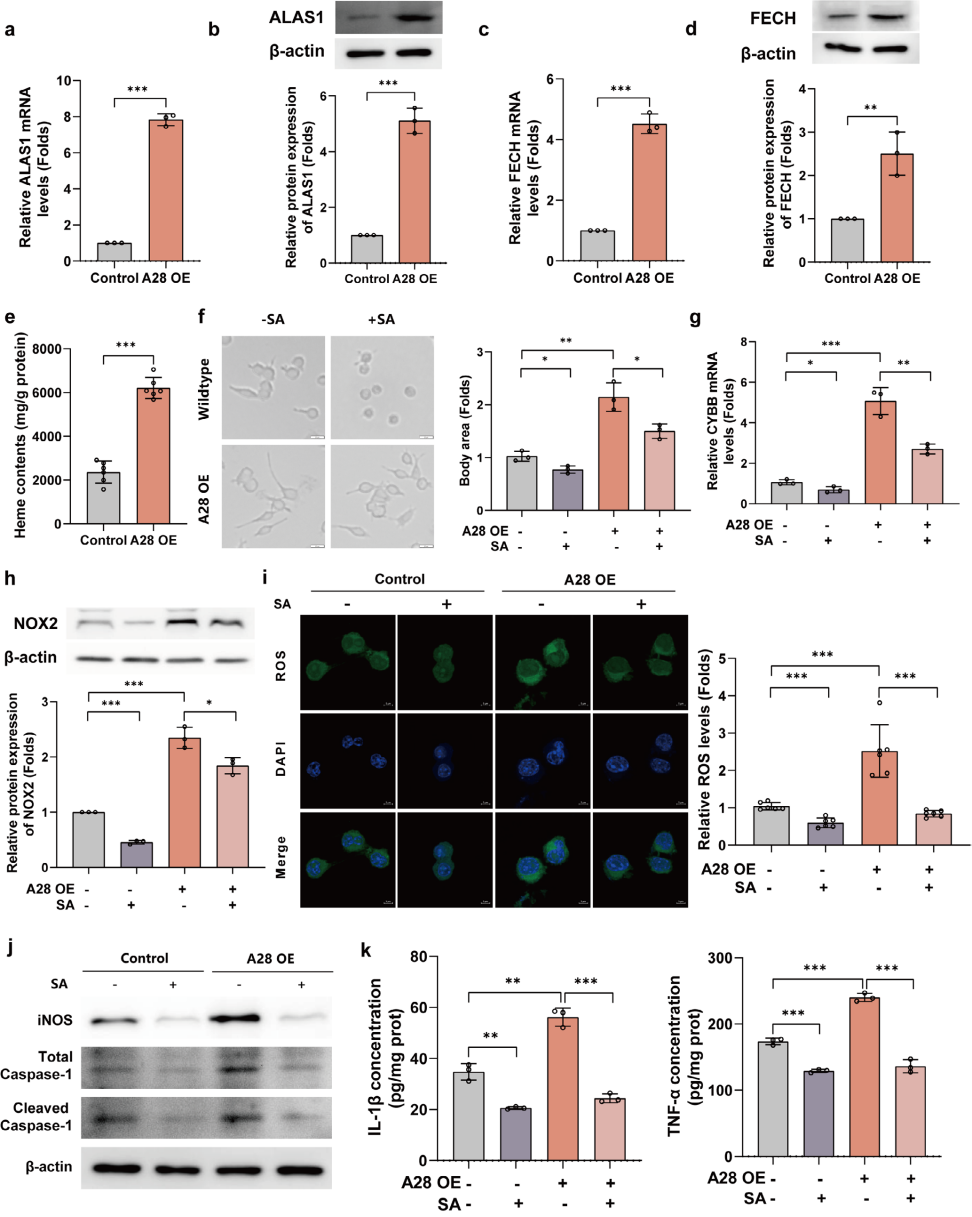
SLC25A28 overexpression promotes NOX2 activation through enhanced heme biosynthesis. (a) ALAS1 mRNA levels in SLC25A28 overexpression (A28 OE) versus control cells (qPCR, *n* = 3). (b) ALAS1 protein expression in A28 OE and control cells (Western blot, *n* = 3). (c) Ferrochelatase (FECH) mRNA levels in A28 OE and control cells (qPCR, *n* = 3). (d) Ferrochelatase protein expression in A28 OE and control cells (Western blot, *n* = 3). (e) Cellular heme content in A28 OE cells (Oxalate assay, *n* = 6). (f) Microglial morphology in A28 OE cells with or without succinylacetone (SA) treatment (scale bar = 20 μm, *n* = 3). (g) NOX2 mRNA levels in A28 OE cells with or without SA treatment (qPCR, *n* = 3). (h) NOX2 protein expression in A28 OE cells with or without SA treatment (Western blot, *n* = 3). (i) ROS production in A28 OE cells with or without SA treatment (DCFH‐DA staining, scale bar = 5 μm, *n* = 6). (j) Inflammatory marker expression in A28 OE cells with or without SA treatment (iNOS, total caspase‐1, and cleaved caspase‐1; Western blot, *n* = 3). (k) ELISA quantification of IL‐1β and TNF‐α levels in A28 OE cells with or without SA treatment (*n* = 3). Data are expressed as mean ± SEM. *p < 0.05, **p < 0.01, ***p < 0.001.

Given that NOX2 activity is critically dependent on heme incorporation for its catalytic function [18] and mitochondrial iron serves as a key substrate for heme biosynthesis [19], we hypothesized that SLC25A28‐mediated mitochondrial iron accumulation drives NOX2 activation by enhancing heme synthesis. In SLC25A28‐overexpressing BV2 cells, both mRNA and protein levels of ALAS1, the rate‐limiting enzyme in heme synthesis, were significantly upregulated (Figure 6a,b), along with increased expression of ferrochelatase (FECH), which catalyzes the final iron insertion step in heme biosynthesis (Figure 6c,d). Oxalate assay confirmed a 2.6‐fold increase in cellular heme content (Figure 6e), directly linking SLC25A28 overexpression to heme overproduction. Morphological analysis revealed that A28 OE microglia exhibited a pronounced activated phenotype, characterized by enlarged cell bodies and ramified processes, which was markedly attenuated by the heme biosynthesis inhibitor succinylacetone (SA) (Figure 6f).

To establish the causal role of heme in NOX2 activation, SA treatment completely abolished A28 OE‐induced NOX2 upregulation, reducing both mRNA and protein levels to baseline (Figure 6g,h). ROS detection further demonstrated that SA eliminated the oxidative burst caused by A28 OE (Figure 6i). Critically, SA reversed the pro‐inflammatory effects of A28 OE, as evidenced by reduced expression of iNOS, total caspase‐1, and cleaved caspase‐1 (Figure 6j). This anti‐inflammatory effect was further validated by ELISA assays, which showed that SA treatment significantly decreased IL‐1β and TNF‐α secretion in A28 OE cells (Figure 6k), consistent with the Western blot results.

These findings mechanistically connect SLC25A28‐mediated mitochondrial iron transport to NOX2 activation through a heme‐dependent pathway, providing the first evidence that mitochondrial iron metabolism in microglia regulates neuroinflammation by supplying enzymatically active heme to NADPH oxidases.

### 3.6 Restoration of Heme Synthesis Reverses the Anti‐Inflammatory Effects of SLC25A28 Knockout

**FIGURE 7:**
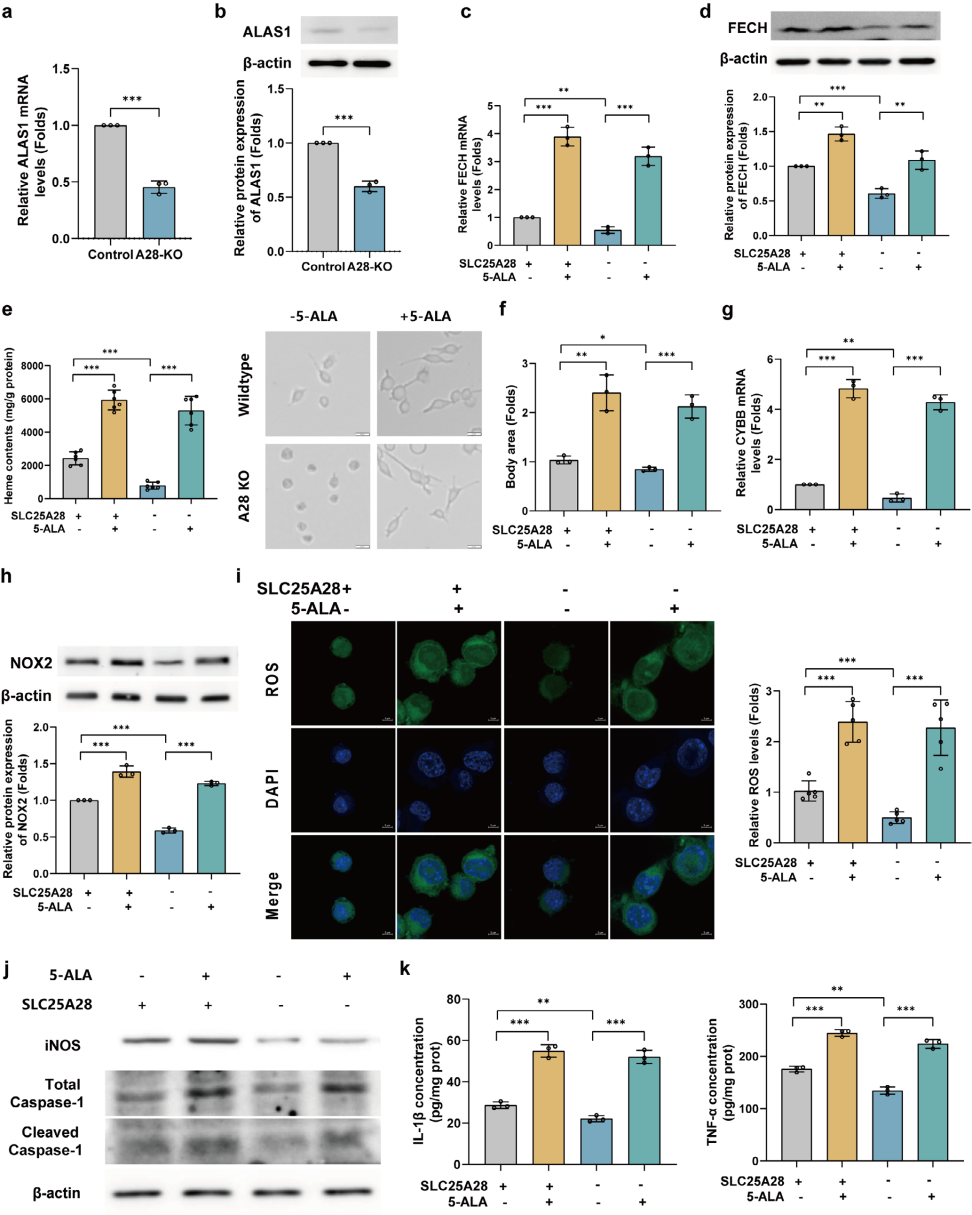
SLC25A28 knockout attenuates neuroinflammation through heme‐dependent NOX2 activation. (a) ALAS1 mRNA levels in A28‐KO versus control primary microglia (qPCR, *n* = 3). (b) ALAS1 protein expression in A28‐KO and control microglia (Western blot, *n* = 3). (c) FECH mRNA levels in A28‐KO versus control microglia (qPCR, *n* = 3). (d) FECH protein expression in A28‐KO and control microglia (Western blot, *n* = 3). (e) Cellular heme content in A28‐KO cells (oxalate assay, *n* = 6). (f) Microglial morphology in A28‐KO cells with or without 5‐aminolevulinic acid (5‐ALA) treatment (scale bar = 20 μm, *n* = 3). (g) NOX2 mRNA levels in A28‐KO cells with or without 5‐ALA treatment (qPCR, *n* = 3). (h) NOX2 protein levels in A28‐KO cells with or without 5‐ALA treatment (Western blot, *n* = 3). (i) ROS production in A28‐KO cells with or without 5‐ALA treatment (DCFH‐DA staining, scale bar = 5 μm, *n* = 5). (j) Inflammatory marker expression in A28‐KO cells with or without 5‐ALA treatment (iNOS, total caspase‐1, and cleaved caspase‐1; Western blot, *n* = 3). (k) ELISA quantification of IL‐1β and TNF‐α secretion in A28‐KO cells with or without 5‐ALA treatment (*n* = 3). Data are expressed as mean ± SEM. *p < 0.05, **p < 0.01, ***p < 0.001.

To confirm that SLC25A28 knockout (A28‐KO) attenuates neuroinflammation by suppressing heme‐dependent NOX2 activation, we performed rescue experiments in A28‐KO primary microglia using 5‐aminolevulinic acid (5‐ALA), a heme precursor that bypasses the ALAS1‐dependent rate‐limiting step. A28‐KO microglia exhibited significantly reduced expression of ALAS1 (Figure 7a,b) and FECH (Figure 7c,d), along with a 59% decrease in heme content (Figure 7e). Morphological analysis revealed that A28‐KO microglia displayed a less activated phenotype, characterized by smaller cell bodies and fewer processes, which was reversed by 5‐ALA supplementation (Figure 7f). 5‐ALA restored FECH expression (Figure 7c,d) and significantly increased heme levels (Figure 7e), thereby reactivating the NOX2 pathway, as evidenced by increased NOX2 mRNA (Figure 7g) and protein levels (Figure 7h), along with elevated ROS production (Figure 7i). Importantly, 5‐ALA reversed the anti‐inflammatory phenotype of A28‐KO, reversing the suppression of iNOS, total caspase‐1, and cleaved caspase‐1 (Figure 7j). Consistent with these findings, ELISA analysis further confirmed that 5‐ALA treatment restored the secretion of pro‐inflammatory cytokines IL‐1β and TNF‐α in A28‐KO microglia (Figure 7k), reinforcing the essential role of heme in mediating SLC25A28‐dependent neuroinflammatory responses. These results conclusively demonstrate that SLC25A28‐KO mitigates neuroinflammation by limiting heme availability for NOX2 activation, with heme synthesis serving as an indispensable downstream mechanism.

## 4 Discussion

Spinal cord injury (SCI) induces a secondary injury cascade dominated by neuroinflammation, with microglial activation contributing substantially to the pathological process. While microglia are essential for debris clearance and early repair, prolonged activation promotes oxidative stress, cytokine release, and tissue degeneration [20, 21]. It is widely accepted that microglial reactivity contributes significantly to the progression of SCI pathology, but the metabolic mechanisms underlying this response remain incompletely understood. Previous studies have largely attributed microglial activation to external inflammatory signals and nonspecific metabolic stressors; the role of defined intracellular metabolic regulators remains poorly understood [22]. This study highlights the role of the mitochondrial iron transporter SLC25A28 in controlling microglial activation and neuroinflammation following SCI.

Mitochondrial iron has recently emerged as a critical modulator of immune cell function, particularly in microglia [23]. Mitochondrial iron overload increases oxidative phosphorylation in early activation and contributes to ROS production via Fenton chemistry and enzymes like NADPH oxidase [24]. Prior work in models of neurodegeneration has shown that mitochondrial iron accumulation contributes to microglial activation and neurotoxicity through elevated ROS and inflammasome signaling [23, 25]. Our time‐course analysis showed that mitochondrial iron levels in spinal cord tissues peaked on Day 3 after injury, coinciding with the highest level of microglial activation. This iron accumulation was closely associated with increased expression of the mitochondrial iron transporter SLC25A28, whereas SLC25A37 expression did not follow the same temporal trend. These findings suggest that SLC25A28 plays a distinct role in regulating mitochondrial iron in SCI, reflecting that mitochondrial iron metabolism varies by cell type and pathological context [26].

The causal role of SLC25A28 in SCI pathology was confirmed using a tamoxifen‐inducible, microglia‐specific knockout model. Mice lacking SLC25A28 in microglia showed improved motor recovery, reduced edema, and better histological preservation compared to controls. These protective effects are consistent with prior reports that limiting microglial activation confers neuroprotection in acute CNS injury [27]. Importantly, our data extend these observations by showing that protection arises through disruption of mitochondrial iron accumulation, which suppresses oxidative stress and inflammasome activation. Supporting this, in vitro studies demonstrated that SLC25A28‐deficient microglia exhibited reduced ROS generation and lower pro‐inflammatory cytokine expression. Together, these findings establish SLC25A28 as a critical upstream regulator of the microglial inflammatory state. Furthermore, the pronounced neuroprotection in A28‐MGKO mice (evidenced by enhanced neuronal survival via Nissl staining, Figure 3f; preserved ultrastructure via TEM, Figure 3h) likely reflects not only reduced microglial pro‐inflammation but also improved microglia–neuron metabolic crosstalk, an aspect tied to mitochondrial iron's role in regulating cellular metabolism. Our data show SLC25A28 knockout mitigates microglial mitochondrial iron overload (Figure 4a–c), and prior studies link such iron dyshomeostasis to disrupted microglia–neuron metabolic communication: excessive mitochondrial iron triggers microglial metabolic reprogramming (e.g., dysregulated glycolysis) and ferroptotic stress, leading to neurotoxic byproducts or inflammatory cascades that disturb neuronal energy homeostasis [28, 29, 30]. By alleviating this iron overload, A28‐MGKO may restore beneficial metabolic crosstalk to support neuronal survival. Future metabolic profiling studies should define how altered microglial iron metabolism disrupts microglial–neuronal crosstalk in the injured spinal cord, elucidating the basis of the knockout outcomes.

Building on evidence that mitochondrial iron contributes to both chemical and enzymatic ROS production, we turned our attention to NOX2, a major ROS‐producing enzyme in microglia. Its activity depends on heme, whose synthesis relies on mitochondrial iron availability [31, 32]. Prior studies have shown that NOX2‐derived ROS is a major driver of neuroinflammation in multiple CNS disorders, and heme availability directly controls its catalytic function [33]. We build upon these insights by showing that SLC25A28 overexpression enhances heme biosynthetic enzyme expression (ALAS1 and FECH), increases cellular heme content, and upregulates NOX2 activity and ROS production in microglial cells. These effects were reversed by pharmacological inhibition of heme synthesis, confirming that the pro‐inflammatory phenotype induced by SLC25A28 is heme‐dependent. These results provide the first direct mechanistic link between mitochondrial iron import and NOX2 activation via the heme biosynthesis pathway in microglia. To further validate the heme–NOX2 axis as a downstream effector of SLC25A28, we performed rescue experiments using 5‐aminolevulinic acid (5‐ALA), which bypasses the ALAS1‐dependent rate‐limiting step in heme biosynthesis. In SLC25A28‐deficient microglia, 5‐ALA supplementation restored heme production, reactivated NOX2, and reinstated ROS generation and pro‐inflammatory cytokine expression. These results reinforce the conclusion that intracellular heme availability is essential for NOX2 activation in microglia.

The role of heme in neuroinflammation has often been studied in the context of extracellular heme release following hemorrhage or trauma, where it contributes to oxidative damage and induces compensatory anti‐inflammatory responses, such as HO‐1 expression [34]. In contrast, our findings emphasize the importance of de novo heme biosynthesis within microglia as a prerequisite for NOX2‐dependent ROS production. Unlike extracellular heme, which may act as a damage signal, intracellularly synthesized heme functions as a structural cofactor required for the assembly and enzymatic activity of NOX2. This distinction highlights a unique mechanism whereby mitochondrial iron import via SLC25A28 supports pro‐inflammatory signaling, not by direct ROS generation, but by enabling enzymatic ROS production through heme‐dependent NOX2 activation. These findings underscore the importance of considering the source, localization, and functional role of heme in shaping inflammatory responses in the CNS.

Beyond NOX2, mitochondrial iron‐derived ROS can also activate NF‐κB, a master regulator of pro‐inflammatory cytokines, and promote NLRP3 inflammasome assembly [35, 36, 37]. Thus, SLC25A28 may integrate heme‐NOX2‐dependent ROS production with canonical inflammatory signaling, situating this axis within the broader network of SCI‐induced neuroinflammation. This connection highlights that targeting SLC25A28 could dampen multiple inflammatory cascades (NOX2, NF‐κB, NLRP3) to support neuroprotection, aligning our findings with existing research.

Taken together, our findings identify SLC25A28 as a promising therapeutic target for modulating microglial inflammation after SCI. By reducing mitochondrial iron accumulation in microglia, SLC25A28 inhibition may offer neuroprotection while minimizing systemic effects on iron homeostasis. Nevertheless, several limitations should be noted. Different SCI paradigms (e.g., contusion/compression vs. transection) exhibit distinct inflammatory dynamics and lesion characteristics [27, 38], which may influence the activation scale and functional impact of the SLC25A28–heme–NOX2 pathway. Our current work focused on a clip‐compression model; future studies should therefore test the generalizability of this mechanism across injury types. Furthermore, given our primary focus on the acute phase post‐injury, it remains unclear whether the neuroprotective effects mediated by SLC25A28 persist into the subacute and chronic recovery stages. Future studies should thus extend observations to the subacute phase to clarify the long‐term durability of these effects. Finally, as our experiments were limited to male mice, potential sex differences in microglial iron metabolism and inflammatory responses merit investigation.

Beyond SCI, mitochondrial iron dysregulation and oxidative stress are also implicated in traumatic brain injury, stroke, Alzheimer's disease, and Parkinson's disease [39, 40]. Given its role in regulating iron import, heme biosynthesis, and NOX2 activity, SLC25A28 may contribute more broadly to microglial dysfunction and neuroinflammation in these disorders. Determining whether the SLC25A28–heme–NOX2 axis represents a convergent mechanism across neurological diseases will require further study. In addition, the development of selective pharmacological inhibitors targeting SLC25A28 could open new therapeutic avenues for modulating microglial metabolism and inflammation in CNS injury and neurodegeneration.

## 5 Conclusions

In summary, our study delineates a novel pathway whereby SLC25A28‐mediated mitochondrial iron transport facilitates heme biosynthesis and NOX2‐dependent ROS generation, thereby driving microglial activation following spinal cord injury. This pathway elucidates a mechanistic link between mitochondrial iron dysregulation and microglial inflammatory responses, positioning SLC25A28 as a critical regulatory node. These findings enhance our understanding of neuroinflammation in SCI and underscore the therapeutic potential of targeting SLC25A28.

## Author Contributions

H.C. and Sc.G. contributed equally to this work as co‐first authors. Conceptualization: H.C. and Sc.G.; methodology: H.C., Sc.G., Y.X., Y.M., and T.P.; software: Y.M.; validation: H.C., R.H., Y.X., Y.M., T.P., L.F., W.L., and R.M.; formal analysis: H.C., R.H., and Y.X.; investigation: H.C., R.H., Y.X., Y.M., T.P., L.F., W.L., R.M., B.Y., Y.Z., and L.L.; resources: Sp.G. and J.Y.; data curation: H.C., R.H., and Y.X.; writing – original draft: H.C. and R.H.; writing – review and editing: H.C., R.H., J.Y., and Sp.G.; visualization: Y.M. and L.L.; supervision: Sp.G. and J.Y.; project administration: Sp.G.; funding acquisition: Sp.G. and J.Y.

## Ethics Statement

The animal study protocol was approved by the Biomedical Ethics Committee of the Health Science Center of Xi'an Jiaotong University (Protocol No. XJTUAE 2023‐2166). All experimental procedures strictly adhered to the ARRIVE guidelines (Animal Research: Reporting In Vivo Experiments). Animals were randomly assigned to each experimental group to ensure unbiased results.

## Conflicts of Interest

The authors declare no conflicts of interest.

## Supporting information


**Figures S1–S2:** cns70638‐sup‐0001‐FigureS1‐S2.docx.

**Table S1:** cns70638‐sup‐0002‐TableS1.docx.

## Data Availability

All data generated or analyzed during this study are included in this published article and its Figures S1 and S2 and Table S1. Additional requests can be directed to the corresponding author.
