# Supplementary material for: Microglial SLC25A28 Knockout Mitigates Spinal Cord Injury in Mice by Inhibiting Heme Synthesis and Subsequent NOX2 Activation
Source: CNS Neurosci Ther. 2025 Nov 2;31(11):e70638. doi: 10.1111/cns.70638 (PMC12580241; doi:10.1111/cns.70638)

Supplementary Figure 1

Fig. 1 Single-cell transcriptomic profiling reveals dynamic microglial activation after spinal cord injury (SCI). UMAP visualization of cell populations in the uninjured spinal cord and at 1, 3, 7, and 14 days post-SCI.


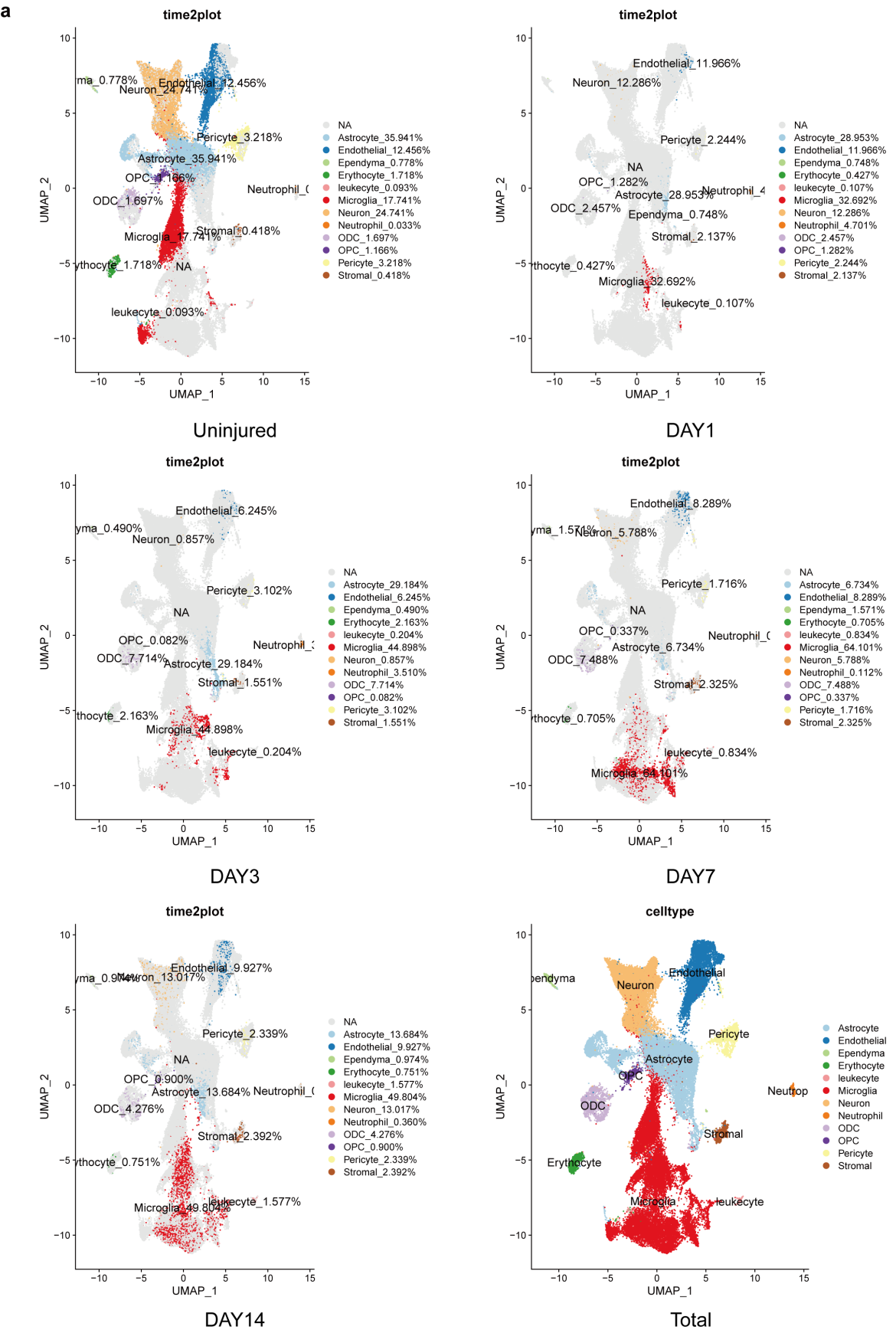


Supplementary Figure 2

Fig. 2 Schematic diagram of the experimental strategy for microglia-specific SLC25A28 knockout induced by tamoxifen


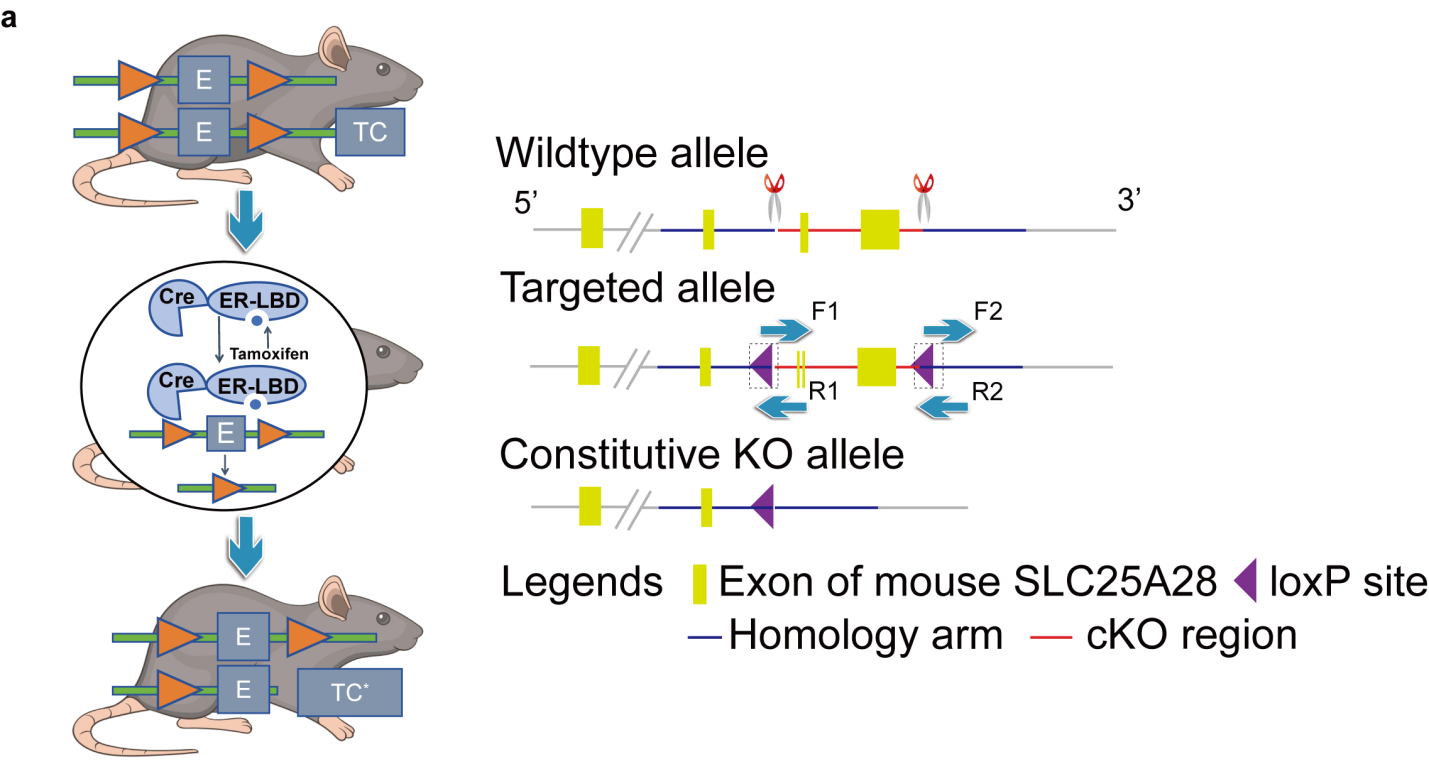

Supplement: Supplementary file 1 — Figures S1–S2: cns70638‐sup‐0001‐FigureS1‐S2.docx. [file CNS-31-e70638-s002.docx]
