# Supplementary material for: Microglial SLC25A28 Knockout Mitigates Spinal Cord Injury in Mice by Inhibiting Heme Synthesis and Subsequent NOX2 Activation
Source: CNS Neurosci Ther. 2025 Nov 2;31(11):e70638. doi: 10.1111/cns.70638 (PMC12580241; doi:10.1111/cns.70638)
Supplement: Supplementary file 2 — Table S1: cns70638‐sup‐0002‐TableS1.docx. [file CNS-31-e70638-s001.docx]

***Supplementary Table***

Microglial SLC25A28 knockout mitigates spinal cord injury in mice by inhibiting heme synthesis and subsequent NOX2 activation

Huangtao Chen, Shaochun Guo, Yanglan Mi, Ruili Han, Yuxin Xi, Tingwei Peng, Longhui Fu, Weidong Liu, Ruiyu Ma, Beibei Yu, Yongfeng Zhang, Luyao Li, Jing Ye * and Shouping Gong *

***** Correspondence: shpingg@126.com (S.G.); yejing1219@gmail.com (J.Y.)

| **Gene Symbol** | **Forward Primer (5'-3')** | **Reverse Primer (5'-3')** |
| --- | --- | --- |
| ***Slc25a28*** | AGCATTGCGTGATGTACCCG | CCTGTTGCTGTGACGTTCA |
| ***Slc25a37*** | CCTACTCCACGATGCAGTAATG | AGTGAATTGACTGGAAGGGGATA |
| ***Cybb*** | TGTATCTGTGTGAGAGGCTGGTG | TGTATCTGTGTGAGAGGCTGGTG |
| ***Alas1*** | TCGCCGATGCCCATTCTTATC | GGCCCCAACTTCCATCATCT |
| ***Fech*** | CAGACAGATGAGGCTATCAAAGG | CACAGCTTGTTGGACTGGATG |
| ***18S rRNA*** | ATGGCCGTTCTTAGTTGGTG | CGCTGAGCCAGTCAGTGTAG |
| ***Actb*** | Ggctgtattcccctccatcg | ccagttggtaacaatgccatgt |

**Supplementary Table 1.** Primer pairs for quantitative PCR.
